# Neuromodulation for Lower Urinary Tract Dysfunction – An Update

**DOI:** 10.1100/tsw.2007.173

**Published:** 2007-06-22

**Authors:** Zahid Hussain, Simon C. W. Harrison

**Affiliations:** Department of Urology, Pinderfields General Hospital, Wakefield, WF1 4DG, UK

**Keywords:** bladder, neuromodulation, urinary incontinence, urinary retention, interstitial cystitis, electric stimulation

## Abstract

The aim of this review is to provide an update on the use of neuromodulation using sacral nerve stimulation for the treatment of disorders of the lower urinary tract. Neuromodulation using the InterStim® system (Medtronic Inc.) is now accepted as an established therapeutic option for patients with detrusor overactivity, and for women with retention or severe voiding difficulties. However, the use of nerve stimulation in modulating lower urinary tract function has to be regarded as a technique that is in its infancy. Much has yet to be learned about the mechanism by which neuromodulation exerts its effects and there is a need to better define the clinical indications for the treatment. There is also work to be done in terms of optimising stimulation delivery, both in anatomical and electronic terms.

## INTRODUCTION

Sacral neuromodulation has been available as a treatment option for patients with lower urinary tract disorders for more than a decade (Table 1). More than 25,000 patients have received InterStim® therapy worldwide for urge incontinence, frequency/urgency, urinary retention in women, chronic constipation, and faecal incontinence[1]. A number of prospective trials and numerous case series have provided an evidence base that has confirmed the efficacy and durability of the treatment[2]. However, substantial gaps in our knowledge remain and this review will look at some of the questions that have been raised during the first 14 years of clinical experience with sacral nerve stimulation in the management of lower urinary tract disorders.

## HOW DOES NEUROMODULATION WORK?

The mechanism of action for neuromodulation is complex and not fully understood. This is not surprising as the pathophysiological mechanisms that underlie the conditions that it is used to treat, including idiopathic detrusor overactivity and urinary retention in women, are themselves not fully understood.

**TABLE 1**
**Milestones for InterStim Therapy**

| | |
|---|---|
| Dec 1993 | Multicentre clinical studies of InterStim therapy began in the U.S. |
| April 1994 | InterStim therapy was commercially released in Europe, Canada, and Australia. |
| Sept 1997 | FDA approved InterStim therapy for the treatment of urinary urge incontinence. |
| April 1999 | FDA approved InterStim therapy for the treatment of urinary retention and significant symptoms of urgency-frequency. |
| Feb 2002 | FDA approved the revised InterStim therapy indication to include the term “overactive bladder”. |
| Sept 2002 | FDA approved a minimally invasive lead implant technique, using the InterStim tined lead (Models 3093/3889). |
| June 2004 | The U.K. National Institute for Clinical Excellence approved sacral nerve stimulation as a treatment option for urgency and urge incontinence. |

However, evidence supports an emerging consensus that it exerts its effect through afferent pathways that modulate the activity in other neural pathways within the spinal cord and higher centres[3]. The evidence for an afferent-pathway mechanism for neuromodulation comes from a variety of different experimental techniques, some of which will be outlined in the following paragraphs.

Studies measuring the delay in motor response during percutaneous nerve evaluation (PNE) of the S3 foramina suggest that complex neural systems rather than direct efferent stimulation are involved in the process of neuromodulation[4]. A concentric needle electrode was used to measure latency of anal sphincter contraction in nine women undergoing PNE prior to implant for either urinary retention or detrusor overactivity. A mean latency of 98 msec (range 52–140 msec) was observed. The authors would have expected much shorter latencies if the response had been due to direct stimulation of the pudendal nerve (3–5 msec). However, it remained unclear whether the response was due to a segmental reflex or involved spino-bulbo-spinal pathways.

A similar study was performed in three patients with complete spinal cord injury presenting with neurogenic detrusor overactivity and detrusor sphincter dyssynergia[5]. This report describes two responses after S3 stimulation: a constant and reproducible early response with a mean latency of 41 msec (33–62 msec) corresponding to a segmental reflex latency, and a more variable late response at a minimum of 183 msec. As in the study by Fowler et al.[4], no direct motor response was observed. These findings indicate that spinal pathways may well be involved in producing the anal and pelvic floor response to S3 stimulation since the spinal cord injury will have interrupted the spino-bulbo-spinal pathways. However, the authors noted no improvement in incontinence episodes or urodynamic parameters with test stimulation, suggesting that the pathways involving supraspinal structures are important for successful neuromodulation. In their opinion, this would therefore exclude patients with complete spinal cord injury from neuromodulatory intervention for their bladder dysfunction.

Further evidence that stimulation of pudendal afferent pathways is responsible for the beneficial neuromodulatory effect is provided by studies of dorsal penile nerve stimulation[6]. Kirkham et al. used an acute study methodology to demonstrate improvements in bladder capacity and compliance in spinal cord injury patients (complete and incomplete) with neurogenic detrusor overactivity. This was achieved by stimulation of the dorsal penile nerve, a purely sensory nerve, using cutaneous electrodes.

Several studies have investigated the effects of neuromodulation on the higher centres. Braun et al. performed serial electroencephalograms (EEG) in 10 patients with both neurogenic and idiopathic bladder dysfunction who had permanent S3 neuromodulation implants (Medtronic, InterStim 3023)[7]. They demonstrated electrical cortical activity in the postcentral sensory gyrus. EEG recordings were made whilst the stimulation from the neuromodulator was switched on and off for 20 cycles. They found that reliable maximum potential amplitudes were recorded by electrodes close to the postcentral gyrus, supporting the potential role of suprasacral centres in neuromodulation.

Blok et al. used positron emission tomography (PET) to study the brain effects during both chronic and acute sacral neuromodulation in patients with urge incontinence[8]. Each patient underwent 10 scans with their neuromodulator switched on for only five of them (random order). In the eight patients who had their S3 neuromodulator first switched on in the PET scanner, they found activity in areas involved in sensorimotor learning. In the 10 patients implanted for more than 6 months, there was activity in areas they have previously shown to be involved in detrusor overactivity, awareness of filling, urge, and the timing of micturition.

Dasgupta et al. also used PET imaging in a controlled study to demonstrate that supraspinal centres are likely to be involved in neuromodulation for urinary retention due to sphincter overactivity in women[9]. A control group of eight healthy volunteers showed enhanced brainstem and cortical activity in response to a full bladder. However, the study group (eight patients) showed only cortical activity with the stimulator off, whereas the brain stem activity was noted with the stimulator switched on. This demonstrated that there is an abnormal interaction between the brainstem and cortex in the study group. In addition, voiding function appeared to be restored by the neuromodulatory effect of resetting brainstem function and reducing cortical activity.

## INDICATIONS FOR AND RESULTS OF NEUROMODULATION

Neuromodulation has offered a completely new approach to managing lower urinary tract dysfunction. It is clear that it is a versatile therapy, but the extent of the indications for which it may be applied is not yet established. There are also difficulties in assessing the proportion of patients who will derive significant long-term benefit from implantation of a neuromodulator.

The use of mean changes in symptom scores and objective measures, such as pad usage and catheterisation frequency, is appropriate when the aim of a study is to prove an effect compared to placebo (phase II studies). However, the impact that a treatment might have when introduced into clinical practice is better gauged by a number needed to treat analysis. In other words, clinicians need to be clear as to what proportion of patients receiving a neuromodulator implant will receive substantial benefit over a medium and long-term period. In order to provide this information, an assessment is needed as to what outcomes will equate to a clinically valid, substantial benefit. Many studies provide a strong indication of success through measures such as zero pad usage for incontinence patients and no requirement for intermittent catheterisation in cases of retention. However, reports in the literature have often included the use of a 50% improvement in one or more main parameters as equating to significant improvement. The question remains as to whether patients with improvement, but not cure, are really representative of success. An unequivocal definition of success would probably include a combination of quality of life and objective measures in addition to a minimum period of follow-up. Until such measures become standardised, our appreciation of the clinical impact of neuromodulation will be incomplete.

A further issue that arises when trying to interpret different studies is that of the relationship between neuromodulation benefit and symptom severity. Cost pressures in many health care systems will effectively ration the use of sacral nerve stimulation and will tend to mean that the technique is mainly used in patients with severe symptoms. It is important that long-term, follow-up data are collected in order to establish if reserving this treatment for the patient with more severe symptoms represents an appropriate strategy.

The following sections look at some of the data that exist in relation to the use of neuromodulation for different indications.

### Bladder Overactivity

Modulation of afferent pathways is believed to inhibit sacral interneuronal transmission, thereby blocking signals from the bladder to the pontine micturition centre[10]. This theory extends to include an explanation of why voluntary micturition is unaffected by sacral neuromodulation.

An important systematic review by Brazzelli et al. looked at the evidence base in relation to urge incontinence[2]. They reported a continence rate (defined as a more that 90% improvement in incontinence symptoms) of 50% in patients having InterStim therapy compared to 1.6% in controls with urge incontinence in randomised controlled trials after 6 months follow-up. The corresponding cure rate in case series was 39% (range 7–64%). However, benefit short of complete continence was seen in other patients, which meant that 67% of patients in case series achieved at least a 50% improvement in incontinence symptoms. The review also looked at adverse events. The most common complication was pain at the implant site (25%). A 33% (282 of 855 patients) surgical revision rate is reported; the most common indications were pain at the implant site, lead migration, and infection. Permanent explantation took place in 9% of 514 patients. The authors commented on the suboptimal quality of trials available for analysis.

It is to be hoped that the continued evolution of the technique, better patient selection, and increased experience will improve results, thereby reducing the relevance of data from earlier studies. However, such an improvement cannot be taken for granted. The Cleveland Clinic Foundation has presented its complications data using a two-stage implantation technique over the period July 2002 to September 2004[11]. They proceeded to the second stage implant in 161/212 patients (75%). The indications for implantation were refractory urge-frequency or urge incontinence (68%), urinary retention (17.4%), and interstitial cystitis (14.6%). The explantation rate was 10.5% due to either infection (5%) or failure to maintain effect (5.5%), while the patient revision rate was 16%. It seems clear that patients must continue to be carefully counselled regarding possible adverse events and failure to maintain a satisfactory long-term response to therapy.

## Urinary Retention in Women

The mechanism by which sacral neuromodulation restores bladder function in patients with idiopathic urinary retention remains unclear. Leng and Chancellor suggest that the positive effect is due to modulation of an abnormal guarding reflex[10]. This excitatory reflex involves activation of sympathetic efferents to urethral smooth muscle and is activated in response to an increase in bladder pressure. In contrast, Fowler et al. have suggested that many women in retention exhibit an abnormal pattern of muscular activity in the external sphincter, which may be due to a primary abnormality of the sphincter rather than being the result of abnormal reflex activity[12]. They point to PET evidence to suggest that changes in brain activity might underlie the therapeutic benefit of neuromodulation[9].

Aboseif et al. studied long-term efficacy and complications of sacral neuromodulation in patients with idiopathic urinary retention[13]. After successful PNE (32 patients underwent PNE), 20 patients (17 female, three male) were implanted. All patients performed clean intermittent self-catheterisation (CISC), having failed all other forms of therapy. Preimplant urodynamics showed no detrusor activity in any patient. They found that 18 patients (90%) were able to void without the need of CISC, including one patient who could only void with bilateral implants switched on. The remaining two patients improved, but continued to use CISC. The mean voided volume increased from baseline (48 to 198 ml) and the postvoid residual decreased from 315 to 60 ml. They observed a 30% complication rate consisting of seroma formation that resolved spontaneously in three patients, superficial infection managed with antibiotics and wound care in one patient, and mechanical failure in two patients.

Case series results bear out the conclusions from a multicentre, prospective, randomised controlled study that confirmed the efficacy of InterStim therapy in patients with refractory urinary retention[14]. The 37 patients who received early implantation were compared to 31 patients who received implants after 6 months (control group). The primary endpoint of residual volume was significantly higher in the control group at 6 months follow-up. Significantly more patients continued to require catheterisation in the control group (81 vs. 40%). Clinical improvement was maintained throughout the 18-month period after implantation. With inactivation of the neuromodulator, the voiding diary parameters returned to baseline values, indicating that the underlying cause of retention was not cured by treatment.

An interesting question in this field is whether the condition of urinary retention in women can be subdivided into those with demonstrable changes in the urethral sphincter (high resting closure pressures and abnormal EMG activity) and those without these features; the former group have classical Fowler's syndrome and may respond particularly well to sacral nerve stimulation. This suggestion was made in a study of 26 women with Fowler's syndrome who showed an early success rate of 96% (25/26 patients)[15]. At a mean follow-up of 37 months, 20 patients were still voiding spontaneously (77%). The patient revision rate was 54% (21 procedures in 14 patients). The positive effect was maintained up to 5 years after implantation.

De Ridder et al. provided further support for the suggestion that EMG changes are of prognostic value[16]; 82 women in retention underwent PNE and 62 received a permanent S3 implant (76%). After a mean follow-up of 43 months, 21/30 patients with Fowler's syndrome (70%) had maintained efficacy compared to 13/32 patients with idiopathic retention (41%); the difference in response rate proved to be statistically significant. Unfortunately, external urethral sphincter EMG is not used routinely in clinical practice to differentiate these two groups of patients and therefore limits the impact of this finding, although urethral pressure profilometry may also have a role in identifying patients with the condition. Furthermore, in clinical practice, the success rate in the non-Fowler's group may still be felt to justify therapy given the severity of these patients' symptoms.

## Idiopathic Urinary Retention in Young Men

There are very limited data available for this group of patients. There are small numbers of men included in some series that report results of the treatment of urinary retention, but their outcomes are often unclear; studies generally mention the inclusion of male subjects, but tend not to report separate data due to the small numbers involved. Anecdotal reports of efficacy with neuromodulation do exist and our own experience supports the conclusion that the technique can be considered in a highly selected group of men with urinary retention. For example, we have implanted devices in the exceptionally rare situation where a young man presents in retention and has video-urodynamic features of detrusor-sphincter dyssynergia in the absent of any neurological abnormality (unpublished data).

## Interstitial Cystitis

Interstitial cystitis (IC) is a chronic illness characterised by painful inflammation of the bladder. A number of aetiological theories exist, of which damage to the protective glycosaminoglycan layer is widely supported. The underlying cause is unknown. Different phases of the condition have been described, progressing from suspicion, through early and disease phases, to the final phase in which fibrosis has resulted in a contracted bladder[17]. This description helps to explain the heterogeneity of the patient population. The lack of a reliable tool to separate patient groups within the spectrum of disease makes evaluation of new technologies problematic. The National Institute of Diabetes, Digestive and Kidney Diseases (NIDDK) criteria for the diagnosis of IC have been adopted as the basis for clinical studies[18].

Maher et al. indicated that neuromodulation may have a role in the management of IC when they reported on the short-term effect of temporary stimulation[19]. PNE was performed on 15 women with intractable IC in a prospective study. The NIDDK criteria were used to define the study population. The mean duration of symptoms was 5 years, and a variety of oral and intravesical treatments had previously failed. Statistically significant improvements in all measured parameters were observed including bladder pain, voided volume, frequency, nocturia, urgency, and quality of life. No complications were reported during the 7- to 10-day test period. Two patients did not have a significant improvement in both pain and frequency, and a further two patients did not have improvement in frequency. The remaining 11 patients (73%) received S3 neuromodulator implants.

Peters and Konstandt provided further, albeit limited, evidence for a therapeutic effect in IC, demonstrating reduced narcotic requirements in patients with refractory IC who had been implanted with Medtronic neuromodulators[20]. This retrospective study of 21 patients (17 female, four male) assessed long-term efficacy in IC patients with at least 6 months follow-up. A mean of six previous failed treatments for IC was documented. The decision to implant these patients was based on improvement in frequency and urgency rather than pain criteria. The diagnosis of IC was based on cystoscopic findings after hydrodistension. The mean anaesthetic bladder capacity in this group was 740 ml. The primary endpoint of morphine dose equivalents decreased from 81.6 to 52.0 mg/day ($p$ = 0.015). Although four patients stopped using all narcotics after implant, three patients showed increased requirements. However, on a subjective 7-point scale, no patient reported worsening of their pain symptoms. Almost all of the patients (95%) stated that they would undergo implantation again.

A more robust study by Comiter demonstrated the safety and efficacy of neuromodulation in a prospective study of 25 patients with refractory IC[21]. The diagnosis was based on NIDDK criteria in patients that had failed behavioural modification, pharmacotherapy, and hydrodistension. Assessment included voiding log, pain diary, Interstitial Cystitis Symptom Index, and Interstitial Cystitis Problem Index. A 50% reduction in frequency and nocturia, pain, and a 50% increase in voided volume were used to select patients for permanent implant; on that basis, 17 patients went forward to receive a stimulator. The mean follow-up in the 17 patients with permanent implants was 14 months. There was a statistically significant improvement in all study parameters, including average number of voids per 24 h (mean ± sd, 16.9 ± 4.6 vs. 8.4 ± 3.5), nocturia (4.5 ± 2.7 vs. 1.7 ± 1.6), voided volume (111 ± 45 vs. 264 ± 102 ml), and pain score on a scale from 0 to 10 (5.8 ± 2.2 vs. 1.6 ± 1.5). There were also statistically significant improvements in both the Interstitial Cystitis Symptom Index and Problem Index. There were no reported complications with either test stimulation or permanent implantation.

## OPTIMUM STIMULATION DELIVERY

There are many unanswered questions in relation to the evolving field of neuromodulation. In particular, we are not yet sure as to how to deliver optimal electrical stimulation for any individual patient with a particular condition. The current Medtronic system can be used at the S3 or S4 levels, and stimulation can be applied unilaterally or bilaterally. Furthermore, optimum programming strategies are not well understood. The device is extremely flexible so that programming can alter the electrode configuration (producing different anatomical sites of stimulation within the foramen and different patterns of current flow), pulse width, frequency, amplitude, and periodicity of stimulation. Furthermore, stimulation parameters can be set up on the basis of either sensory responses (perianal or vaginal perception of stimulation) or on motor effects (anal, pelvic floor, and toe responses). Consideration may also be given to the waveform that is used to stimulate nerves. In the context of intravesical stimulation, investigators have compared saw tooth rather than square wave pulses[22]. These are areas that are ripe for further research.

One area that has received attention is that of the possible benefit of using bilateral rather than unilateral stimulation. The theoretical benefits of permanent bilateral stimulation are that patients showing a partial response to unilateral stimulation may show improvement and that lower stimulation parameters may be required with bilateral stimulation resulting in longer battery life. However, there is a significant cost implication involved in this technique. Scheepens et al. compared unilateral vs. bilateral percutaneous nerve evaluation in a prospective randomized crossover trial in 33 patients with a variety of chronic voiding dysfunctions[23]. Radiographs were used to check position and monitor for lead migration at the start and end of the 10-day study and resulted in exclusion of eight patients. In the subgroup of urge-incontinent patients (n = 12), there was no significant difference in pad usage, number of voids per 24 h or volume per void. In the retention subgroup (n = 13), there was no difference in volume per void or catheterized volume. However, two patients began to void with bilateral stimulation only. They received a bilateral implant (Medtronic Synergy 7427) with durable response at 6 months follow-up. Thus, only urinary retention was identified as a possible predictor for bilateral stimulation. The authors suggest that a bilateral PNE be considered if a unilateral test fails. In our practice, bilateral PNE is performed if the placement of a single lead results in a suboptimal motor response. This may direct the location of a permanent implant if the test stimulation is successful. Unfortunately, there are no large prospective studies investigating the role of bilateral permanent stimulation.

## SHOULD TWO-STAGE IMPLANTATION REPLACE THE PNE?

Janknegt et al. first described the technique of two-stage implantation using a permanent electrode (PICE-QUAD, Medtronic) fixed to the sacrum with sutures, as an alternative to the standard PNE for selecting patients for implantation of the full Medtronic system[24]. They hypothesised that this technique would help to differentiate nonresponders from technical failures, given that movement of the PNE wire within the foramen was a significant problem at that time. Furthermore, a longer test period was available for assessment as the implanted lead was attached to a tunnelled extension cable and external pulse generator. A carefully selected group of 10 patients who had failed two PNEs, but shown a good initial response (acute phase), were considered for the two-stage implant. Indications for neuromodulation included urge incontinence (four patients), retention (four patients), and frequency/urgency (two patients). Eight patients responded with more than 50% improvement and went on to have a successful implant. One of the failures had had a S4 electrode test and when this was changed to S3, she had a successful first-stage implant. Therefore, only one patient with retention failed to respond the new chronic testing phase. The report of long-term results was limited. In the urge-incontinent group, pad usage at 6 months follow-up had decreased from 7.2 to 0.4 per day.

With the development of the tined lead and the minimally invasive implantation technique, it became possible to perform the first stage under local anaesthesia. Spinelli et al. demonstrated an 80% positive response rate to test stimulation using this technique in patients who did not have a prior PNE[25]. The study population included three male and 15 female patients with a variety of voiding dysfunction including idiopathic and neurogenic overactivity, retention and frequency/urgency. These results were maintained in all patients after the second stage of implantation. These results further opened up the question as to whether there is a significant false-negative rate from the PNE test that is reduced by using the two-stage methodology; typical reported permanent implant rates after PNE are 60% compared with the 80% reported by Spinelli et al.

Borawski et al. compared PNE to first-stage lead placement (FSLP) using the tined lead in a prospective randomised trial[26]. They studied 30 female patients aged >55 years with refractory urge incontinence. Both procedures were performed under local anaesthesia. There was no difference in pain scores after the test stimulation procedure. In the PNE group, 6/13 (46%) had a positive test compared to 15/17 in the FSLP group (88%). The difference was felt to be of such significance that the study was closed after an interim analysis before recruiting the planned 35 patients. The long-term outcomes in the implanted patients are awaited. If both groups were to maintain their clinical effect equally, the superiority of the two-stage technique would be proven in this population of neuromodulatory subjects and the PNE would potentially be replaced by routine two-stage implantation. Indeed, it could even be argued that all suitable patients should simply receive the full implant, given that the principle reason for not going straight to implantation is cost; the cost of the implants that do not prove successful would be offset by the savings on the additional hospital costs that the two-stage implant generates.

## NOVEL NEUROMODULATION TECHNIQUES

The commercial development of the InterStim system by Medtronic has pushed neuromodulation into the realm of a viable therapy that can be offered to appropriate patients. However, other stimulation systems are also being examined and developed.

A novel surgical technique for implanting a new electrostimulation system has been described for women with bladder overactivity[27]. This involves the placement of a paraurethral electrode and a suprapubic electrostimulator (Miniaturo TM, Biocontrol Medical Ltd, Yehud, Israel). It has been evaluated in seven women with frequency, urgency, and urge incontinence over a period of 12 months. Assessment was made of voids per 24 h, degree of urgency (graded 0 to 3), leakage episodes, and pad usage. The King's College Hospital incontinence questionnaire was used to measure the change in quality of life (QoL). Descriptive analysis of their results is shown in Table 2. Urodynamic studies showed that only one patient had idiopathic detrusor overactivity and her bladder capacity increased from 30 to 177 ml before leakage. One patient developed an infection, requiring explantation of the electrostimulator after 10 months.

**TABLE 2**
**Results**

| | Baseline | 12 months |
|---|---|---|
| Voids/24 h | 15.2 (12.0–21.3) | 9.4 (6.0–13.0) |
| Degree of urgency | 2.0 (1.2–3.0) | 1.4 (0–2.6) |
| Leaking episodes | 9.1 (4.3–15.0) | 1.8 (0–6.7) |
| Pad usage | 7.8 (2.0–15.7) | 1.5 (0–5.0) |
| QoL | 43.6 (35.0–53.0) | 16.3 (9.0–31.0) |

The authors recommended that all patients should have a staged procedure and undergo external test stimulation before permanent implantation. The complete procedure could be performed under local anaesthesia. They postulated that the positive effect of stimulation of pelvic floor muscles was due to inhibition of the micturition reflex. This preliminary study has demonstrated efficacy. Safety data are available from a larger group of implanted patients for a variety of indications including IC[28]. Adverse events occurred in 34/79 patients, requiring surgical intervention in 29 patients (37%). In some cases, multiple surgical interventions were required.

These promising results with this new minimally invasive procedure indicate that further evaluation of this system is needed in the form of prospective controlled studies. In December 2005, the Miniaturo received European CE mark approval for IC and the overactive bladder syndrome. In April 2006, the company sold its urology line of products to American Medical Systems together with an exclusive license for use of its electrical stimulation technology for treatment of pelvic floor conditions in the fields of urology and gynaecology[29].

Interest has also focussed on the possibility of stimulating the pudendal nerve rather than the segmental S3 nerve. A neurosurgical mapping study of pudendal nerve afferents has shown that asymmetrical distribution exists in most patients[30]. The relative contributions from S1, S2, and S3 were 4, 60.5, and 35.5%. However, the afferent distribution was sometimes confined to a single level (18%) and even to a single root (7.6%). This wider representation of stimulation levels and possible stimulation of the most relevant afferent fibres might give pudendal nerve stimulation an advantage over S3 foraminal stimulation.

A novel technique involves pudendal nerve stimulation within Alcock's canal using a self-contained, battery-powered, programmable microstimulator with an integrated electrode (Bion therapy, Advanced Bionics Corporation, Valencia, CA)[31]. This device measures 27 × 3.3 mm and weighs 0.7 g. A percutaneous stimulation test is performed under local anaesthesia, using sensory responses to optimise localisation. This is an acute test, as evaluation is by cystometrogram after 10 min of stimulation. The test is considered positive if there is either 50% increase in bladder capacity or a 50% increase in bladder capacity at first involuntary contraction. Permanent implantation is through a 3-mm skin incision medial to the ischial tuberosity and can be performed with sedo-analgesia. Thus, the implant can be positioned using both sensory and motor responses.

A pilot study evaluated the efficacy of this technique in 14 women with refractory detrusor overactivity and incontinence[32]. After testing, six women received a permanent implant (43%). There was a significant improvement in incontinence episodes as well as improvement in average voided volumes and bladder capacity at urodynamic testing. No severe adverse events were reported. The authors have commented on the short testing period, suggesting that a longer test may increase the number suitable for permanent implant. In June 2004, Advanced Bionics Corporation was acquired by Boston Scientific Corporation. It has been CE marked in Europe for urinary urge incontinence[33].

Spinelli et al. have also been looking at pudendal stimulation, but using a traditional implanted lead and pulse generator (Medtronic)[34]. They have recently reported preliminary results in 55 patients with encouraging results, but definitive data are awaited.

## References

1. http://www.medtronic.com/physician/interstim/index.html
2. Brazzelli, M., Murray, A., and Fraser, C. (2006) Efficacy and safety of sacral nerve stimulation for urinary urge incontinence: a systematic review. *J. Urol*. **175**, 835–841.
3. Craggs, M. and McFarlane, J. (1999) Neuromodulation of the lower urinary tract. *Exp. Physiol.* **84**, 149–160.
4. Fowler, C.J., Swinn, M.J., Goodwin, R.J., Oliver, S., and Craggs, M. (2000) Studies of the latency of pelvic floor contraction during peripheral nerve evaluation show that the muscle response is reflexly mediated. *J. Urol*. **163**, 881–883.
5. Schurch, B., Reilly, I., Reitz, A., and Curt, A. (2003) Electrophysiological recordings during the peripheral nerve evaluation (PNE) test in complete spinal cord injury patients. *World J. Urol*. **20**, 319–322.
6. Kirkham, A.P.S., Shah, N.C., Knight, S.L., Shah, P.J.R., and Craggs, M.D. (2001) The acute effects of continuous and conditional neuromodulation on the bladder in spinal cord injury. *Spinal Cord* **39**, 420–428.
7. Braun, P.M., Baezner, H., Seif, C., Boehler, G., Bross, S., Eschenfelder, C.C., Alken, P., Hennerici, M., and Juenemann, P. (2002) Alterations of cortical electrical activity in patients with sacral neuromodulator. *Eur. Urol*. **41**, 562–567.
8. Blok, B.F., Groen, J., Bosch, J.L., Veltman, D.J., and Lammertsma, A.A. (2006) Different brain effects during chronic and acute sacral neuromodulation in urge incontinent patients with implanted neurostimulators. *BJU Int*. **98**, 1238–1243.
9. Dasgupta, R., Critchley, H.D., Dolan, R.J., and Fowler, C.J. (2005) Changes in brain activity following sacral neuromodulation for urinary retention. *J. Urol*. **174**, 2268–2272.
10. Leng, W.W. and Chancellor, M.B. (2005) How sacral nerve stimulation neuromodulation works. *Urol. Clin. North Am*. **32**, 11–18.
11. Hijaz, A., Vasavada, S.P., Daneshgari, F., Frinjari, H., Goldman, H., and Rackley, R. (2006) Complications and troubleshooting of two-stage sacral neuromodulation therapy: a single-institution experience. *Urology* **68**, 533–537.
12. Fowler, C.J., Christmas, T.J., Chapple, C.R., Parkhouse, H.F., Kirby, R.S., and Jacobs, H.S. (1988) Abnormal electromyographic activity of the urethral sphincter, voiding dysfunction, and polycystic varies: a new syndrome? *BMJ* **297**, 1436–1438.
13. Aboseif, S., Tamaddon, K., Chalfin, S., Freedman, S., Mourad, M.S., Chang, J.H., and Kaptein, J.S. (2002) Sacral neuromodulation in functional urinary retention: an effective way to restore voiding. *BJU Int*. **90**, 662–665.
14. Jonas, U., Fowler, C.J., Chancellor, M.B., Elhilali, M.M., Fall, M., Gajewski, J.B., Grunewald, V., Hassouna, M.M., Hombergh, U., Janknegt, R., van Kerrebroeck, P.E., Lylcklama a Nijeholt, A.A., Siegel, S.W., and Schmidt, R.A. (2001) Efficacy of sacral nerve stimulation for urinary retention: results 18 months after implantation. *J. Urol*. **165**, 5–9.
15. Dasgupta, R., Wiseman, O.J., Kitchen, N., and Fowler, C.J. (2004) Long-term results of sacral neuromodulation for women with urinary retention. *BJU Int*. **94**, 335–337.
16. De Ridder, D., Ost, D., and Bruyninckx, F. (2007) The presence of Fowler's syndrome predicts successful long-term outcome of sacral nerve stimulation in women with urinary retention. *Eur. Urol*. **51**, 229–233.
17. Kurth, K.H. (2004) *Interstitial Cystitis*. Ergebnisse Verlag. pp. 14–16.
18. National Institute of Diabetes, Digestive and Kidney Diseases (1994) Interstitial Cystitis. NIH Publication No. 94-3220. U.S. Department of Health and Human Services, Public Health Service, National Institutes of Health, Rockville, MD.
19. Maher, C.F., Carey, M.P., Dwyer, P.L., and Schluter, P.L. (2001) Percutaneous sacral nerve root neuromodulation for intractable interstitial cystitis. *J. Urol*. **165**, 884–886.
20. Peters, K.M. and Konstandt, D. (2004) Sacral neuromodulation decreases narcotic requirements in refractory interstitial cystitis. *BJU Int*. **93**, 777–779.

21. Comiter, C.V. (2003) Sacral neuromodulation for the symptomatic treatment of refractory interstitial cystitis: a prospective study. *J. Urol*. **169**, 1369–1373.
22. De Bock, F., DeWachter, S., Van Immerseel, L., and Wyndaele, J.J. (2007) Exploring the use of triangular waveform for intravesical electrical stimulation. *Eur. Urol. Suppl.* **6**, 140.
23. Scheepens, W.A., de Bie, R.A., Weil,E.H., and van Kerrebroeck, P.E. (2002) Unilateral versus bilateral sacral neuromodulation in patients with chronic voiding dysfunction. *J. Urol*. **168**, 2046–2050.
24. Janknegt, R.A., Weil, E.H., and Eerdmans, P.H. (1997) Improving neuromodulation technique for refractory voiding dysfunctions: two-stage implant. *Urology* **49**, 358–362.
25. Spinelli, M., Giardiello, G., Gerber, M., Arduini, A., van den Hombergh, U., and Malaguti, S. (2003) New sacral neuromodulation lead for percutaneous implantation using local anesthesia: description and first experience. *J. Urol*. **170**, 1905–1907.
26. Borawski, K.M., Foster, R.T., Webster, G.D., and Amundsen, C.L. (2007) Predicting implantation with a neuromodulator using two different test stimulation techniques: a prospective randomized study in urge incontinent women. *Neurourol. Urodyn.* **26**, 14–18.
27. Nissenkorn, I. and De Jong, P.R. (2005) A novel surgical technique for implanting a new electrostimulation system for treating female overactive bladder: a preliminary report. *BJU Int*. **95**, 1253–1258.
28. Bosch, J.L. (2006) Electrical neuromodulatory therapy in female voiding dysfunction. *BJU Int*. **98(Suppl 1)**, 43–48.
29. http://www.biocontrol-medical.com/about.htm
30. Huang, J.C., Deletis, V., Vodusek, D.B., and Abbott, R. (1997) Preservation of pudendal afferents in sacral rhizotomies. *Neurosurgery* **41**, 411–415.
31. Bosch, J.L. (2005) The bion device: a minimally invasive implantable ministimulator for pudendal nerve neuromodulation in patients with detrusor overactivity incontinence. *Urol. Clin. North Am*. **32**, 109–112.
32. Groen, J., Amiel, C., and Bosch, J.L. (2005) Chronic pudendal nerve neuromodulation in women with idiopathic refractory detrusor overactivity incontinence: results of a pilot study with a novel minimally invasive implantable mini-stimulator. *Neurourol. Urodyn*. **24**, 226–230.
33. http://www.bostonscientific.com/templatedata/imports/HTML/AboutBostonScientific/our_businesses_6.shtml
34. Spinelli, M., Malaguti, S., Citeri, M., Zanollo, L., and Redaelli, T. (2007) Update on chronic pudendal nerve stimulation in sacral area dysfunctions. *Eur. Urol. Suppl*. **6**, 140.
